# Synchronous well differentiated neuroendocrine tumour and gastrointestinal stromal tumour of the stomach: a case report

**DOI:** 10.1186/1471-230X-11-27

**Published:** 2011-03-24

**Authors:** Vassilis D Samaras, Periklis G Foukas, Konstantinos Triantafyllou, Vassilia Leontara, Dimitrios Tsapralis, Eirini M Tsompanidi, Anastasios Machairas, Ioannis G Panayiotides

**Affiliations:** 1 2ndDepartment of Pathology, University of Athens Medical School ("Attikon" University Hospital), (1 Rimini), Chaidari, Athens, (12462), Greece; 2 Gastroenterology Unit, 2ndDepartment of Internal Medicine - Propedeutic, University of Athens Medical School ("Attikon" University Hospital), (1 Rimini), Chaidari, Athens, (12462), Greece; 3 3rdDepartment of Surgery, University of Athens Medical School ("Attikon" University Hospital), (1 Rimini), Chaidari, Athens, (12462), Greece

**Keywords:** stomach, neuroendocrine tumour, GIST

## Abstract

**Background:**

Well differentiated neuroendocrine tumours (carcinoids), arising from cells of the diffuse neuroendocrine system, represent the most commonly encountered gastric endocrine tumours. Gastrointestinal stromal tumours (GISTs), which stem from interstitial Cajal cells located within the wall of the gastrointestinal tract and have a characteristic immunoreactivity for CD117 (c-kit protein), account for the majority of gastrointestinal mesenchymal neoplasms. Simultaneous occurrence of a GIST with a well differentiated neuroendocrine tumour in the stomach is very rare.

**Methods:**

Clinical history, endoscopy and histopathological findings were utilized for our diagnostic considerations.

**Results:**

We report the coexistence of a high risk GIST with a well differentiated neuroendocrine tumour of benign clinical behavior, both located in the stomach, in a 62-year-old man previously operated for a gastric well differentiated neuroendocrine tumour with uncertain malignant behaviour.

**Conclusions:**

Even single well differentiated, sporadic, NETs of small size may coexist with GISTs. An appropriate initial therapeutic approach combined with a scrupulous follow-up seems to play a significant role in terms of preventing a metastatic disease.

## Background

Gastrointestinal neuroendocrine tumours (NETs) are thought to derive from cells of the diffuse neuroendocrine system of the gastrointestinal (GI) tract [1]. In the stomach, most endocrine tumours are located in the corpus or fundus, constituting a group of nonfunctioning, enterochromaffin-like (ECL) cell, well differentiated NETs (carcinoids). Gastric NETs are divided into four types: a) type I, associated with autoimmune chronic atrophic gastritis (A-CAG), b) type II, linked to multiple endocrine neoplasia type 1 (MEN-1) or to the Zollinger-Ellison syndrome (ZES) and c) type III, sporadic, unrelated to hypergastrinemia or A-CAG, d) type IV, representing a heterogeneous group of tumours which show evidence of multidirectional differentiation, such as a combination of adenocarcinoma and NET [2-4].

Gastrointestinal stromal tumours (GISTs) are mesenchymal neoplasms stemming from the interstitial cells of Cajal localized within the wall of the GI tract [5,6]. GISTs show a predilection for the stomach, where they constitute the majority of mesenchymal tumours [7,8]. Immunopositivity for CD117 (c-kit protein) is a distinctive immunohistochemical feature of these tumours [8,9], for which tumour size and mitotic activity are the most significant parameters regarding prediction of clinical behavior [10,11].

GISTs have been reported to coexist with a variety of neoplasms, the percentage of such cases ranging from 4.5% to 33% [12,13]; in such cases, the stomach is the most frequent location of GIST [12]. The most common coexisting tumour is adenocarcinoma of the gastrointestinal tract [12,13]; other types include lymphoma, leukemia, carcinomas of breast, prostate, pancreas or lung or adrenocortical adenoma [12-16]. Similarly, previous studies have shown an association between GI NETs and synchronous or metachronous epithelial tumours accounting for about 10% to 46% of cases [17,18].

What is more, coexistence of GISTs with gastrointestinal tract endocrine tumours, such as ileal well differentiated NET [19] or well differentiated NET of the pancreas [14] has also been reported. However, the simultaneous occurrence of gastric well differentiated NET and gastric GIST seems to be rare, a handful of cases having so far been reported, to our best knowledge [7,9,12,20].

In this context, we hereby present the case of synchronous occurrence of a GIST and a well differentiated NET, both located in the stomach of a male patient. Our report seems to be distinctive as it comprises unique clinical and histopathological findings.

## Case presentation

**Figure 1:**
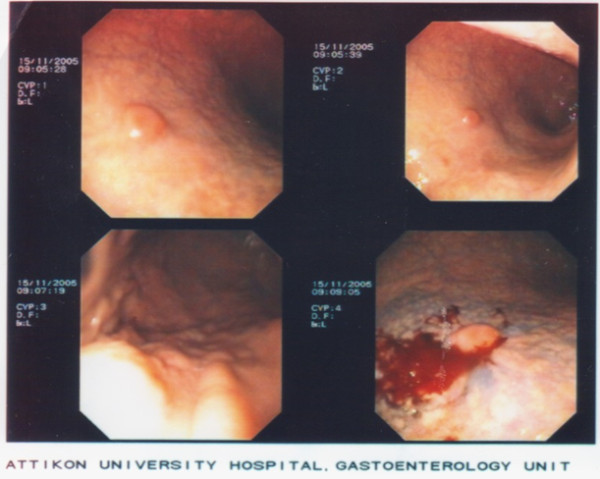
**Gastroscopy; an approximately 1.2 cm large lesion in the greater curvature of stomach**. Note the smooth contour of the mildly protruding lesion as well as its bleeding even with gentle endoscopic manipulations.

A 58-year-old male was admitted for chronic muscular pain. During hospitalization, an episode of melena occurred, following which he underwent both colonoscopy and gastroscopy; the latter disclosed an approximately 1.2 cm large lesion in the greater curvature of stomach [Figure 1]. A proximal subtotal gastrectomy (with subsequent reconstruction of the GI tract), including the lesion, was therefore performed. A 6.5 × 4.2 × 1 cm surgical specimen containing a mildly protruding, 1.2 cm large mucosal area at a distance of 0.8 cm from the closest surgical margin was received; the remaining mucosa was unremarkable. Histology showed the lesion to be a completely excised, well differentiated NET with relatively uniform tumour cells, immunopositive for chromogranin-A, synaptophysin and CD56; no detectable mitoses were seen. The tumour infiltrated both muscularis mucosae and the contiguous part of the submucosa. Proliferation index by means of a Ki67/MIB-1 immunostain was lower than 2%. No vascular emboli were seen. The tumour was therefore classified as a gastric well differentiated NET with uncertain malignant behaviour [2,21-23]. Adjacent gastric mucosa showed mild chronic gastritis, diffuse incomplete intestinal metaplasia of epithelium, as well as endocrine cell hyperplasia of simple, linear or micronodular types. An extensive post-operative work-up showed neither signs of tumour extension, nor serum evidence of autoimmunity; the patient was therefore placed on a regular follow-up. A year later, he underwent another gastroscopy with biopsies showing chronic inactive gastritis with foci of diffuse, both complete and incomplete intestinal metaplasia of the gastric epithelium. Helicobacter pylori microorganisms were not detected by a Giemsa stain.

However, four years post-op, CT showed a well delineated, 3.5 cm large, intramural lesion in the esophagogastric junction. Serum gastrin levels were within normal limits. The patient underwent excision of the remaining part of the stomach; this corresponded to a subtotal gastrectomy (with subsequent reconstruction of the GI tract) specimen with a 35 cm long greater and a 14.5 cm long lesser curvature, respectively, together with a 1.9 cm long duodenal cuff. A stenosis was observed in the incisura angularis; moreover, a protruding, multilobular, 3.5 cm large tumour was located close to the esophagogastric junction, on the anterior wall of the stomach, presenting, upon sections, a whitish color with brownish foci and a fibrous consistency. The remaining gastric mucosa was unremarkable.

**Figure 2:**
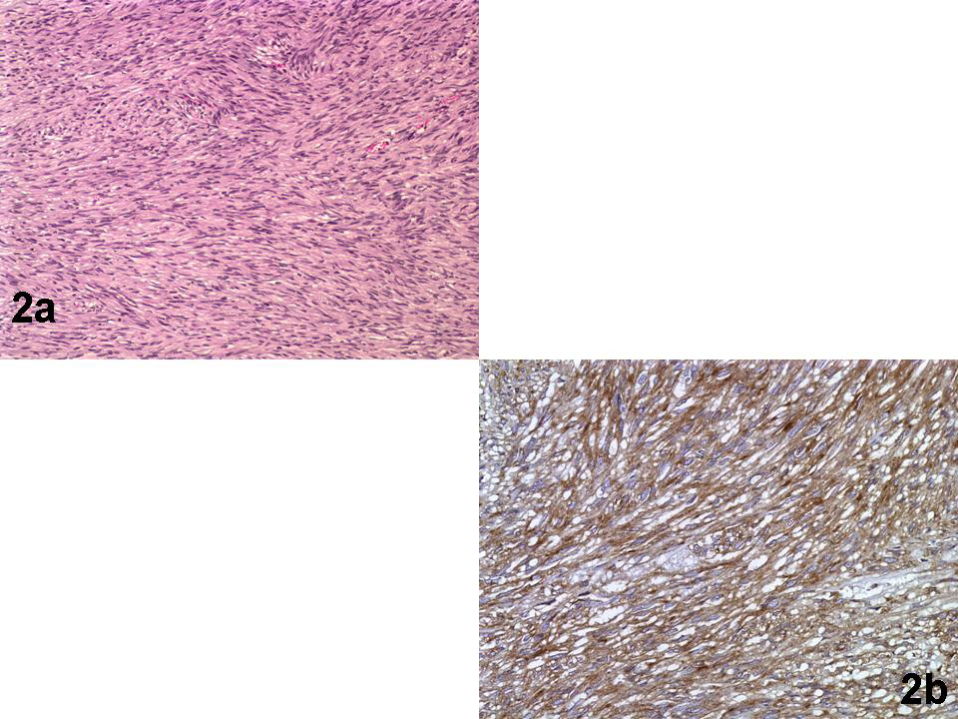
**GIST of the stomach**. Fig.2a (Hematoxylin-Eosin/X10): Neoplastic growth pattern characterized by interlacing bundles of spindle cells with focal mitotic activity. Fig.2b (anti-CD117/X20): Diffuse immunoreactivity of neoplastic cells.

Histology showed the tumour to consist of interlacing bundles of spindle cells [Figure 2]. A total number of 15 mitoses per 50 high power fields (with a ×40 objective) were assessed in the most cellular neoplastic areas. The tumour contained areas of hemorrhagic infiltration or cystic degeneration and had mainly pushing borders. Neoplastic cells were intensely decorated with antibodies against CD117/c-kit [Figure 2], CD34 and vimentin, were focally immunoreactive for α-SMA and S-100 protein, but did not stain for cytokeratin 18. Proliferation index by means of a Ki67/MIB-1 immunostain was approximately 10%, with predominant staining in peripheral tumour areas. Consequently, a diagnosis of moderate risk GIST (based on criteria laid down by the Armed Forces Institute of Pathology) [11] or high risk GIST (based on the National Institute of Health GIST Workshop-2001) [10] was established.

**Figure 3:**
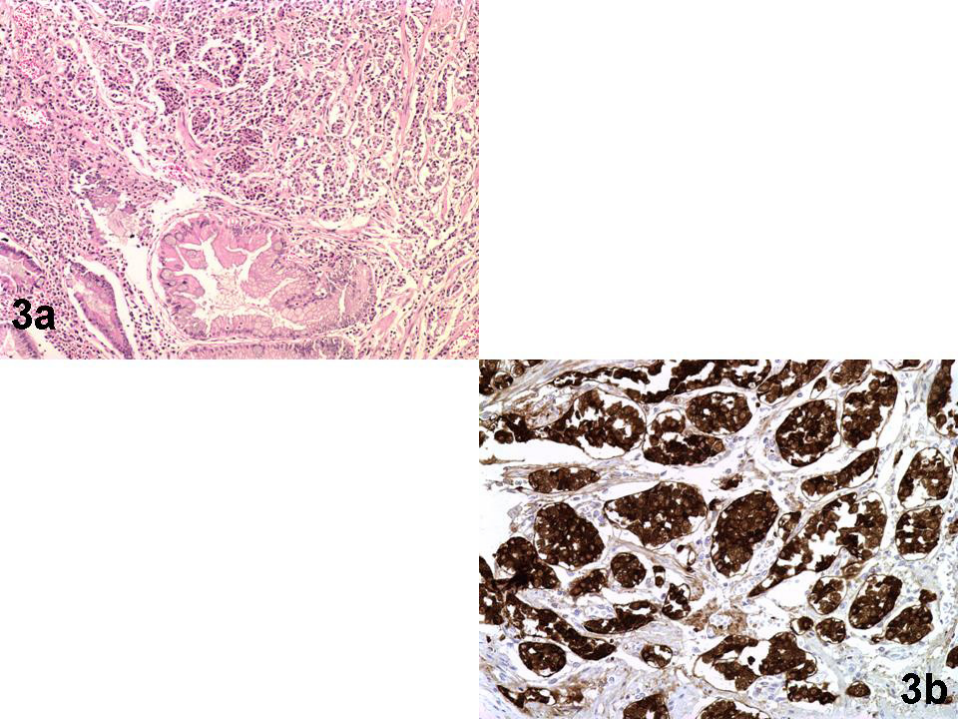
**The second well differentiated neuroendocrine tumour of the stomach**. Fig.3a (Hematoxylin-Eosin/X10): Neoplasm localized in the mucosa and submucosa and consisted of monomorphous cells, with unremarkable mitotic activity, forming adenoid or solid aggregates. Note adjacent gastric glands with areas of intestinal metaplasia. Fig.3b (anti-Chromogranin/X20): Diffuse, intense, immunoexpression by tumour cells.

Moreover, in the stenotic area of the incisura angularis (vide supra), a totally excised, 0.4 cm large, well differentiated NET of benign clinical behavior was identified: it was located in the mucosa and submucosa and consisted of a monomorphic cellular population, with inconspicuous mitotic activity, forming adenoid or solid aggregates [Figure 3]. An Alcian blue stain disclosed no mucin. Tumour cells were immunoreactive for chromogranin-A [Figure 3] and synaptophysin, but not for CD56 or serotonin. The proliferation (Ki-67/MIB-1) index was approximately 2%. No vascular emboli were seen. The adjacent gastric mucosa showed atrophic gastritis along with extensive complete intestinal metaplasia [Figure 3]. Giemsa stain disclosed no Helicobacter pylori. Thus, on the basis of recent data suggesting a role of adjuvant imatinib therapy in terms of improving recurrence-free survival after the resection of primary localized GIST, the patient was placed on imatinib [24]; he is alive and well a year after the second operation.

## Conclusions

Of the previous reports regarding the simultaneous occurence of GIST and well differentiated NET in the stomach we achieved to retrieve data only for three cases. Specifically, one case concerned a 69-year old male with negative family history of cancer who underwent removal of a sessile polypoid mass in the corpus of the stomach. Histological examination of the resected specimen demonstrated a submucosal borderline GIST, while in the overlying mucosa a well differentiated NET (carcinoid), invading the lamina propria and initially infiltrating the submucosa, was recognized. Neither H. pylori microorganisms nor foci of intestinal metaplasia were identified in the surrounding mucosa. The patient died of unrelated cause (heart attack) twelve months after the surgical intervention, while imaging findings were all negative concerning metastatic deposits from the above-mentioned lesions [20].

The second case concerned a 65-year-old female with a low-risk GIST on the anterior wall of the upper gastric corpus concomitant with a type III well differentiated NET localized on the posterior wall of the upper gastric corpus; no recurrence of either tumour occurred 28 months after excision [9]. In the third case a 65-year-old female, who had previously undergone subtotal gastrectomy for a GIST (risk category not mentioned), presented with serum hypergastrinemia and polypoid lesions of the gastric stump, a year post-op. Histology showed polypoid type I gastric well differentiated NETs, while the histopathological examination of the remaining stomach's excision specimen documented the co-existence of a GIST (risk category not mentioned) [7]. Agaimy et al., also, based on their own archives and on data collected from the literature, reported four gastric well differentiated NETs concurrent with GISTs [12].

Regarding pathogenetic mechanisms implicated in the dual development of GIST and well differentiated NET in the stomach, data are still insufficient. Though a role of H. pylori infection cannot be excluded, it is at present not clear, perhaps due to the small number of cases so far reported. Thus, Lin et al. did not conclude any definite causal relationship among GIST, well differentiated NET and H. pylori infection [9], a point also valid in our case, where no H. pylori was shown. The hypothesis which is based on experimental data in rats and supports the notion that a single carcinogenic agent possibly interacts with two neighboring tissues, inducing the development of tumours of different histotypes in the same organ, remains to be substantiated in human tissues [25-27]. In addition, we cannot entirely exclude the possibility that genetic factors concerning specific gene mutations could be responsible for the synchronous development of two gastric tumours. On the other hand, a coincidental growth of two primary neoplasms in the same organ (stomach) could be also a possible consideration. Further studies are certainly needed in order to elucidate this phenomenon.

Gastric well differentiated NETs account for 8.7-41% of all gastrointestinal well differentiated NETs [2]. Their clinical features include abdominal pain, vomiting, anemia, massive gastric bleeding or, rarely, carcinoid syndrome [2,27]. Our patient had an episode of melena preceding the diagnosis of its first well differentiated NET, whereas the second one was incidentally diagnosed.

In our report, both well differentiated NETs were single lesions; despite the fact that the first tumour arose in the context of endocrine cell hyperplasia, the absence of serum autoimmunity indices precludes its classification as a type I gastric well differentiated NET; both tumours, therefore, correspond to type III according to the WHO classification [2-4].

According to the WHO classification, benign behavior of well differentiated NETs is associated with the following criteria: cytologically bland tumour confined to mucosa and/or submucosa, non-angioinvasive, less than 1 cm in size, non-functioning; these features are mainly found in tumours arising in the context of A-CAG or MEN-1/ZES. On the contrary, uncertain malignant behavior of well differentiated NETs is associated with a nonfunctioning, cytologically bland tumour, measuring 1 to 2 cm in diameter, confined to the mucosa and/or submucosa. Angioinvasion may be present in these lesions [2,21-23]. This category comprises some of the type II gastric NETs. Therefore, in our case, the first neuroendocrine tumour was diagnosed with uncertain malignant behaviour due to its size and its being single.

Limited surgical interventions (including endoscopic manipulations) remain the mainstay treatment for type I gastric well differentiated NETs [28]. Partial gastrectomy combined with appropriate treatment for concomitant hypergastrinemia is necessary for type II neoplasms [29]. Finally, a total gastrectomy is counseled for type III well differentiated NETs, even when they are histologically well differentiated, particularly in tumours larger than 1 cm [2,29], a treatment not initially performed in our case, due to the patient's refusal.

GISTs, thought to arise from interstitial Cajal cells of the gastrointestinal wall [5,6], demonstrate no clear gender predilection; affected individuals are mainly adults between the 6th and 8th decade [8]. Gastric GISTs can be incidentally detected during endoscopy, an imaging study or in a surgical specimen, given that they are slowly growing tumours localized within deeper layers of gastrointestinal wall. Though our patient was asymptomatic, GISTs usually manifest with nonspecific symptoms, such as nausea, vomiting, abdominal pain or, most often, with bleeding, due to protrusion of the tumour and stretching of the overlying mucosa; metastasis may sometimes constitute the initial symptom [8,9].

Optimal therapy includes surgical excision and treatment with imatinib. The latter is appropriate for non-operable tumours, cases where total excision is impossible or for recurrences [9,30].

Concerning prediction of clinical behavior, the initial proposal formulated by the National Institute of Health (NIH) GIST Workshop in 2001 defined the risk for aggressive behavior based on tumour size (single largest dimension) and mitotic count (number of mitotic figures per 50 HPF) [10], classifying GISTs into very low, low, intermediate and high risk categories. More recently, the Armed Forces Institute of Pathology (AFIP) suggested a new risk stratification of primary GISTs, introducing the site involved alongside the two criteria previously mentioned, i.e. mitotic index and size [11]; thus, gastric GISTs are stratified as having none, very low, low, moderate or high risk of progressive disease. Regardless of the system used, it should be kept in mind that large tumours (especially those larger than 10 cm), even when having no detectable mitotic activity, may show late recurrences and even metastatic deposits. According to the WHO guidelines, DNA-aneuploidy and high proliferative activity, assessed by proliferation markers, may reflect higher malignant potential [8]. In our case, the GIST was classified as of moderate risk according to the AFIP criteria, whereas it corresponded to the high risk category according to the NIH criteria.

In conclusion, we presented a rare case of a well differentiated NET concurrent with a GIST in the stomach. Gastroenterologists and pathologists, in particular, should be aware of this rare phenomenon given that even single well differentiated, sporadic (unrelated to hypergastrinemia or A-CAG), NETs of small size may coexist with GISTs, as it was shown in our case. A meticulous follow-up (along with a well scheduled initial therapeutic approach) should be performed to all patients suffering from the afore-mentioned neoplasms so as the devastating consequences of a metastatic disease to be avoided.

## Competing interests

The authors declare that they have no competing interests.

## Authors' contributions

All authors have made substantive intellectual contribution to the case report. VDS: Writing of manuscript, conception and design of case report. PGF: Writing of manuscript. KT: Analysis of endoscopic findings. VL: Revising and editing of manuscript, histological diagnosis. DT: Analysis and interpretation of clinical data. EMT: Analysis and interpretation of clinical data. AM: Revising of manuscript. IGP: Revising and editing of manuscript, histological diagnosis. All authors read and approved the final manuscript.

## Pre-publication history

The pre-publication history for this paper can be accessed here:

http://www.biomedcentral.com/1471-230X/11/27/prepub
